# Supplementary material for: Contingency awareness shapes neural responses in fear conditioning
Source: Neurosci Conscious. 2026 Jan 19;2026(1):niaf065. doi: 10.1093/nc/niaf065 (PMC12814967; doi:10.1093/nc/niaf065)
Supplement: Supplementary_v_1_0_niaf065 [file supplementary_v_1_0_niaf065.docx]

# Supplementary

## Pilot study

Experimental design was identical to the experiment reported in the article, except the ISI (time between US presentation and start of the next trial) was 500 ms shorter (1500+/-50 ms).

**Methods**

In the pilot phase of the experiment, we collected a sample of 20 subjects (12 aware and 8 unaware).

As a result of the pilot phase of this study, we expected that about half of the participants would be able to learn the associations between the stimuli (i.e., become contingency aware).

We had several a priori hypotheses.

First, in the test phase if a first category word is presented and left-hand side vibration is expected then contralateral alpha-beta suppression preceding the actual vibration is observed. The same is applicable to the right-hand side and the second category of words.

Second, when expectation is violated e.g. the vibration is delivered to the opposite to the expected hand then error-related negativity (ERN) is generated and SPN precedes US+ presentation.

Third, the expected effects described above would be observed only in the group of aware subjects.

**ROIs**

**ERPs**

SPN1 in 500-900 ms

SPN2 in 1500-2000 ms

SPN ROI: FC1,FCz,FC2,F1,Fz,F2,C1,Cz,C2

**Time-frequency analysis**

Baseline = -0.4 -0.1

*Alpha preceding vibration*

Channels: FC3, FC1, C3, C1, CP3, CP1 (left ROI); FC2, FC4, C4, C2, CP4, CP2 (right ROI)

Time window: 600-900 ms

Frequency band: 8-14 Hz

*Beta preceding US*

Channels: FC3, FC1, FCz, FC2, FC4, C3, C1, Cz, C2, C4

Time window: 1500-2000

Frequency band: 15-22 Hz

**Results**

We did not find support for the first hypothesis. If the word associated with the left-hand side vibration was presented we did not see any lateralization of the alpha-beta suppression preceding the presentation of vibration itself. But there was a non-lateralized effect of alpha suppression in somatosensory cortex in aware group but not in unaware (see Figure S1c. The analyzed ROI is marked by black rectangle). The interaction Group x Condition was not significant, but the effect of Group was (p=0.003).

The second hypothesis was partially confirmed. We did not find anything like ERN in response to the vibrations that violate the learned rule. With that, for SPN_US_, the interaction Group (aware, unaware) x Condition (CS+ - violation of the rule and expectation of US+, CS- - vibration on the expected hand side and expectation of US-) was marginally significant (p=0.098) (see Figure S1a, circled in the right panel). SPN was more negative just before presentation of US+ comparing with expectation of US- in the aware group but not in the unaware group. The effect of Group on SPN_vibro_ (in the time window between word and vibration) presentations was also marginally significant (p=0.100) (see Figure S1a left panel).


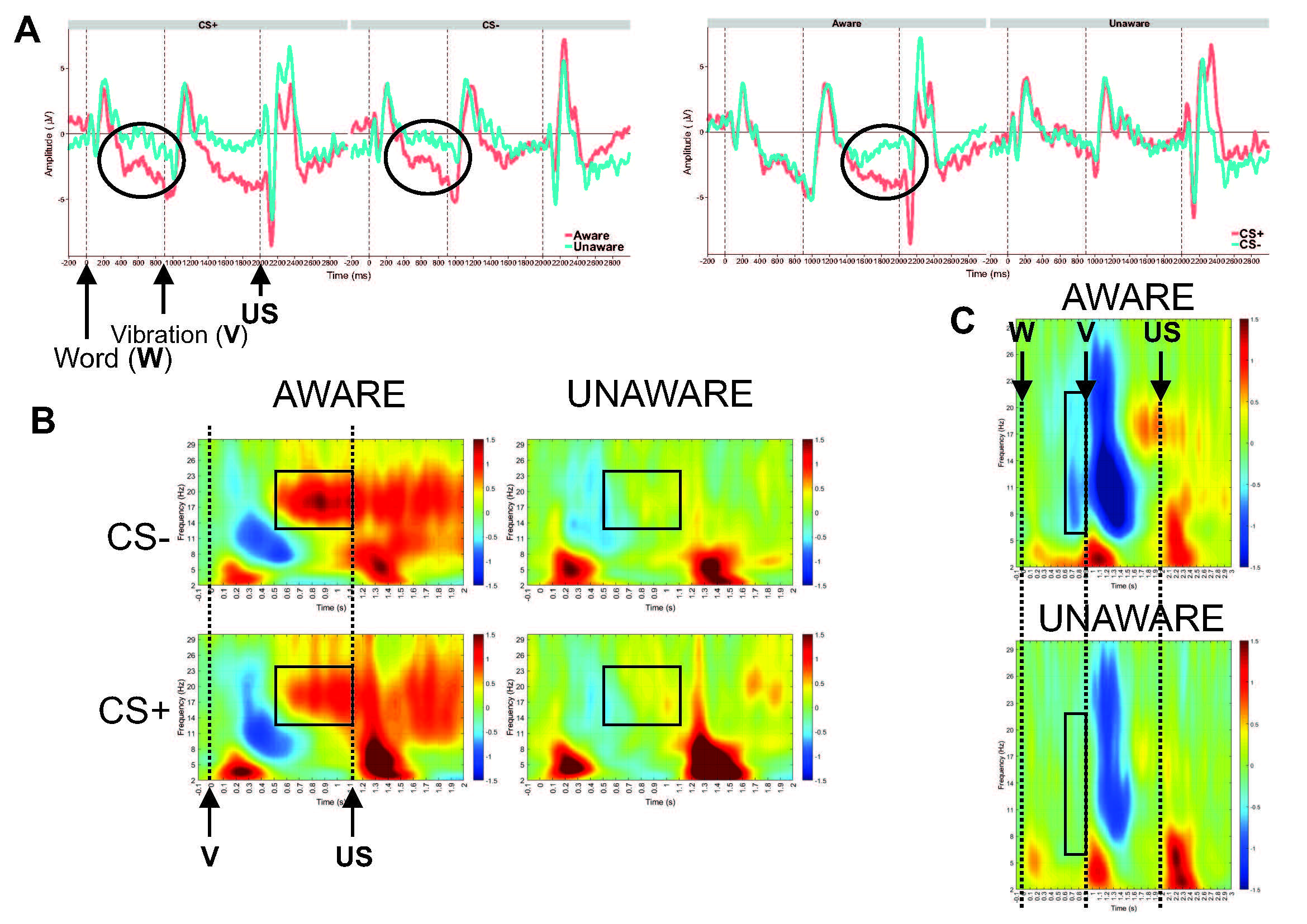


Figure S1 - Summary of the findings in the pilot study. (a) Left panel: Stimulus preceding negativity (SPN) at Cz is significantly different between groups (main effect of Group). Right panel: SPN is more negative in CS+ condition as compared with the CS-, but only in the Aware group (Group x Condition interaction). (b) Group effects on the oscillatory brain activity averaged over FC1, FCz, FC2, C1, Cz, C2 channels in the beta frequency band (the beta rebound effect). (c) Group effects on the alpha activity averaged over C2, C4, C6, CP2, CP4, CP6, FC2, FC4, FC6, C1, C3, C5, CP1, CP3, CP5, FC1, FC3, FC5 channels preceding the vibrations.

**Effect sizes in the pilot study**

N = 20; Group: aware (n = 12) /unaware (n=8); Condition: CS+/CS-

**H1.1, H1.2**

*SPN2 (SPN_US_)*

Channels: FC1,FCz,FC2,C2,C1,Cz,F1,Fz,F2

Time window: 1500-2000 ms after word presentation

Group *F*(1, 18) = 2.77, *p* = .114, $\eta_{p}^{2}$ = .13

Condition *F*(1, 18) = 1.41, *p* = .251, $\eta_{p}^{2}$ = .07

Group x Condition *F*(1, 18) = 3.05, *p* = .098, $\eta_{p}^{2}$ = .15

In the posthoc analysis of simple effects, the effect of condition was found to be significant only in the aware group.

Aware: t(11) = 2.320, p = 0.0323, d = 0.70

Unaware: t(7) = 0.362, p = 0.7214, d = 0.14

**H1.3**

*SPN1 (SPN_vibro_)*

Channels: FC1,FCz,FC2,C2,C1,Cz,F1,Fz,F2

Time window: 500-900 ms after word presentation

Group *F*(1, 18) = 3.01, *p* = .100, $\eta_{p}^{2}$ = .14

Condition *F*(1, 18) = 0.16, *p* = .692, $\eta_{p}^{2}$ < .01

Group x Condition *F*(1, 18) = 0.12, *p* = .732, $\eta_{p}^{2}$ < .01

**H1.4**

*Beta preceding US*

Frequency: 15-22 Hz

Channels: FC3,FC1,FCz,FC2,FC4,C3,C1,Cz,C2,C4,F3,F1,Fz,F2,F4

Time window: 1500-2000 ms after word presentation

Group *F*(1, 18) = 4.18, *p* = .056, $\eta_{p}^{2}$ = .19

Condition *F*(1, 18) = 0.00, *p* = .978, $\eta_{p}^{2}$ < .01

Group x Condition *F*(1, 18) = 0.47, *p* = .501, $\eta_{p}^{2}$ = .03

**H1.5**

*Alpha preceding vibration*

Frequency: 8-14 Hz

Channels: Pz,CPz,CP1,CP2,CP3,CP4,P1,P2,P3,P4

Time window: 500-900 ms after word presentation

Group *F*(1, 18) = 12.11, *p* = .003, $\eta_{p}^{2}$ = .40

Condition *F*(1, 18) = 0.02, *p* = .880, $\eta_{p}^{2}$ < .01

Group x Condition *F*(1, 18) = 0.10, *p* = .754, $\eta_{p}^{2}$ < .01

**H1.6**

*Alpha/beta to vibration*

Frequency: 8-22 Hz

Channels: Left hemisphere: C3,C1,CP1,CP3, Right hemisphere: CP2,CP4,C2,C4

Time window: 1000-1400 ms after word presentation

Group *F*(1, 18) = 4.25, *p* = .054, $\eta_{p}^{2}$ = .19

Condition *F*(1, 18) = 0.01, *p* = .907, $\eta_{p}^{2}$ < .01

Group x Condition *F*(1, 18) = 0.03, *p* = .869, $\eta_{p}^{2}$ < .01

Thus, the smallest Group effect was $\eta_{p}^{2}=$0.13. 75% of it is $\eta_{p}^{2}=$0.0975.

**The analysis of the acquisition phase**


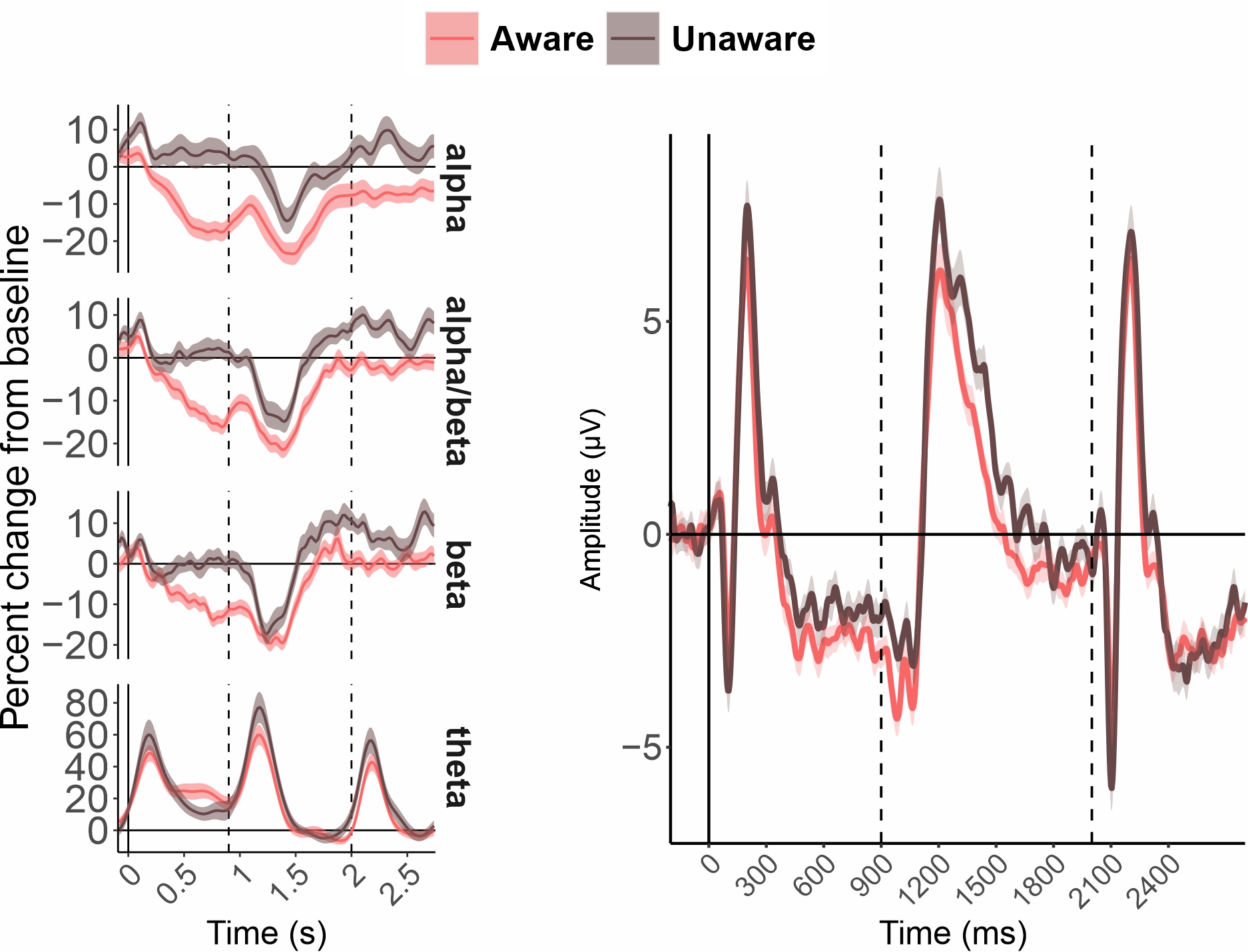


Figure S2 – The results of the analysis of the acquisition phase of the experiment where only CS- trials were presented. The effects in SPN_US_ and SPN_vibro_ time windows did not reach significance.
